# Taxonomy and Identification of the Genus *Scolopendra* in China Using Integrated Methods of External Morphology and Molecular Phylogenetics

**DOI:** 10.1038/s41598-017-15242-7

**Published:** 2017-11-22

**Authors:** Sihe Kang, Yimei Liu, Xiaoxuan Zeng, Haiying Deng, Ying Luo, Keli Chen, Shilin Chen

**Affiliations:** 1 Key Laboratory of Ministry of Education on Traditional Chinese Medicine Resource and Compound Prescription & Hubei University of Chinese Medicine, Wuhan, 430065 P.R. China; 2 Hubei Institute for Drug Control, Wuhan, 430075 P.R. China; 3 Wuhan University of Science and Technology, Wuhan, 430065 P.R. China; 4 Institute of Chinese Materia Medica, China Academy of Chinese Medical Sciences, Beijing, 100700 P.R. China

**Keywords:** DNA sequencing, Taxonomy

## Abstract

The centipede *Scolopendra* has important medicinal value and high toxicity, making it to be an interesting subject for evolutionary studies. However, species identification in China is difficult because of limited resource exploration and lack of recent taxonomic revision. To improve the identification and taxonomy of the genus *Scolopendra* in China, an in-depth investigation was conducted, and an integrated method that combined morphological characteristics with molecular data was applied. The identification key was revised to show the main difference among species. Our results indicated that morphologically-delimited species were consistent with the molecular analysis inferred from the COI sequences with genetic distances and phylogenetic trees. Additional morphometrics of four characteristics provided criteria for shape variation. These results suggested that the members of the genus *Scolopendra* in China could be delineated as 14 separate species. A new species from Lufeng county, Yunnan province, was proposed according to its characteristics, which was named as *S. lufengia* sp. nov. Our results comprehensively ascertained the taxonomic status of *Scolopendra* species in China, explored their phylogenetic relationships, showed a high success in the identification of medicinal centipedes.

## Introduction

The genus *Scolopendra*, which belongs to the family Scolopendridae of the order Scolopendromorpha, is widely distributed across the world, especially in tropical and subtropical territories, such as Southeast Asia^1,2^, North Africa^3^, the Mediterranean^4^, and North or Central America^5,6^. As an ancient biological population, *Scolopendra* is an important branch of terrestrial arthropods in the evolutionary process. Furthermore, populations of many species in *Scolopendra* live in a relatively fixed geographical distribution area due to its weak ability to migrate, so *Scolopendra* has become an important research material for historical biogeography. Reports showed that there were 42 nominal species of *Scolopendra* distributed in the Old World^7^. In the tropical and subtropical areas of southern China, there should be an abundant resource of centipedes^8^. However, resource surveys of the genus *Scolopendra* have rarely been conducted in China. Sporadic evidence has shown that there are 13 species of *Scolopendra* scattered in southern China^8,9^, but the exact species and details of their distribution are still uncertain, and it needs to be revised.

As is well known, centipedes are poisonous, and their venom is highly toxic to vertebrates including humans, which can cause intense pain and edema, sometimes paralysis or even death^10^. However, knowledge about centipede toxicity is still limited. In recent years, studies have targeted the potential therapeutic effects of the toxin^11,12^. After being extracted from centipede venom, a selective NaV1.7 inhibitor was discovered to have analgesic efficacy exceeding that of morphine in rodent pain models^13^. A cytotoxic and anticoagulant peptide was also found in *S. mutilans* venom^14^. In some Asian countries, such as China, some species are even used for antitumoral purposes^15^ or to improve blood rheology^16^. However, it is reported that patients have shown myocardial ischemia due to vasospasm, hypotension, and myocardial toxic effects of the venom^17^. The results of Fang’s study indicated that the histamine content of *S. mutilans* was higher than that of *S. multidens* and *S. mojiangica* ^18^, suggesting that the toxicity of centipedes varies considerably between species. In China, *S. mutilans* L. Koch, 1878, as the dominant and most widespread species, is the only species recorded in Chinese pharmacopoeia as medicinal material^19^. The shape similarity between *Scolopendra* species often causes confusion or misuse, which can pose a large risk to human health as mentioned above. Therefore, it is crucial to improve the accuracy of identification and taxonomy of *Scolopendra* species.

As a universal taxonomic method, morphological characteristics, such as the body size, colour, number of antennal articles, spines on ultimate legs prefemur and tarsal spur on legs, etc., are used in the classification and identification of the genus *Scolopendra*. However, if some species show close relationships or similar characteristics, or some features are missed, it can be challenging to identify these arthropods or obtain a better taxonomic result. To date, the taxonomy and phylogenetic relationships of *Scolopendra* are still controversial, and the validation of the former subspecies of the *S. subspinipes* complex and the *S. morsitans* complex has been debated repeatedly, resulting in their undetermined status^9,20^. Therefore, a simple and accurate method to authenticate the species in the genus *Scolopendra* is urgently needed. The use of morphometrics provides a new pathway for the demarcation of morphological characteristics. The species in Southeast Asia were identified by applying geometric morphometrics data^2^. This method involves the digitization of certain subjective features; thus, the results are more objective and accurate because this method is based on statistical analysis, the potential for human error was prevented as far as possible. However, to have sufficient accuracy, the sampling size should be large enough because a small number of samples or overlapping characteristics could result in confusion. The emergence of molecular phylogeny has led to its use as a powerful supplementary tool in evolutionary studies, as well as biological and medicinal material investigations^21–23^. Based on molecular techniques, the taxonomy and evolutionary history of many organisms have been revised. Currently, COI barcoding technology is the most popular method for the identification of animal species, and it has been widely used on fish^24,25^, birds^26^ and insects^27,28^, as well as centipedes^2^. For instance, the centipede genus *Eupolybothrus* of North Africa had been systematically studied by this method, and a new species, *E. kahfi* Stoev & Akkari, had been discovered^29^. Recently, the integrative methods of morphology combined with DNA data or other analysis were used in the taxonomy of centipedes. Joshi *et al*. used molecular data to identify putative species, and then they applied morphology and ecological niche analysis to reveal the diversity in the Western Ghats of South India^30^. An integrative method including molecular phylogenetics, geometric morphometrics and external morphology was used to delimit seven *Scolopendra* species in mainland Southeast Asia, the former subspecies of *S. dawydoffi*, *S. japonica*, and *S. dehaani* were validated as full species^2^. In the studies of the genus *Scolopendra* in China, in light of preliminary identification of morphology, Zhang^31^ demarcated *S. mutilans* from four other species using COI barcoding. However, the taxonomy and phylogenetic relationships of these species or subspecies still need to be studied further.

In this study, based on an in-depth survey of *Scolopendra* species in the south of China^8^, an integrated method combining morphology with molecular analysis was used to identify and distinguish the species of *Scolopendra* in China. The type materials of some species had been re-described, and morphometric analysis of four critical characteristics were conducted, then an identification key was made. The molecular analysis of COI barcoding was used for further validation of the morphological taxonomy. Furthermore, the taxonomic status of these species and relationships among them were also validated. The works will be instrumental in solving problems related to some centipede species being confused and misused in China.

## Results

### Morphological identification

As described in the methods section, 39 batch samples were observed. The characteristics, i.e, number of antennal articles, presence of gonopods, tarsal spurs on 20th leg and spines on the ultimate leg prefemur, had been identified, and the subtle features were inspected by light microscopy. The diagnostic characteristics of these assigned species are recorded in Table 1. All samples examined had common characteristics of the genus *Scolopendra*. Based on morphological characteristics, the examined samples were divided into seven nominal species and one putative new species. The seven species are as follows: *S. mutilans*, *S. multidens*, *S. dehaani*, *S. mojiangica*, *S. negrocapitis*, *S. subspinipes* and *S. hainanum*. The specimens collected from Lufeng County, Yunnan province, have some characteristics different from others, the details are as follow:

**Table 1:** Characteristics of all examined species based on external morphology of voucher specimens.

Species	*S. mutilans*	*S. multidens*	*S. mojiangica*	*S. negrocapitis*	*S. dehaani*	*S. subspinipes*	*S. hainanum*	*S. lufengia*
Size	Small	Big	Small	Small	Big	Big	Big	Small
Color	D: Cephalic plate and the first tergite fresh red; other tergites dark green	D: Cephalic plate and the first tergites deep red; other tergites brown	M: Blackish green	M: Dark blue	D: Cephalic plate and the first tergite reddish brown; tergites entirely brown. M: Yellowish brown to reddish brown	M: Yellowish brown to dark brown	M: Yellowish brown to dark brown	M: Deep green
Antennal articles	17 – 18	18	18	18	18	18 – 19	17 – 19	18
Teeth	5+5	6+6	4+4 or 5+5	5+5	6+6	5+5 or 6+6	6+6 or 7+7	4+4
Spines on coxopleural process	2	3	2	2 – 3	2	1 – 3	1 – 2	2
Spines on ultimate legs prefemur	2 VL, 1 VM, 1 DM and 2 corner	2 VL, 2 VM, 2 DM and 3 corner	2 – 3 VL, 2 VM, 2 DM and 3 corner	3 VL, 2 VM, 2 – 3 DM and 2 corner	0 VL, 1 VM, 1DM and 3 corner	2 – 3 VL, 1 – 2 VM, 1 – 3 DM and 2 – 3 corner	1VL, 1 VM, 2 DM and 2 corner	1 VL, 2 VM, 2 DM and 3 corner
Tarsal spur on legs	1 – 20	1 – 19	1 – 19	1 – 19	1 – 20	1 – 20	1 – 19	1 – 19
Male gonopods	Present	Absent	Present	Present	Present	Present	Absent	Present
Distribution	Yangzi river system	Zhujiang river system, Sanjiang river (Yunnan) system, Hainan, Taiwan	Yuanjiang river (Yunnan) system	Yangzi river system	Zhujiang river system, Sanjiang river (Yunnan) system, Hainan	Zhujiang river system, Sanjiang river (Yunnan) system, Hainan, Taiwan	Hainan, Guangxi	Lufeng (Yunnan)

VL: ventral lateral; VM: ventral medial; DM: dorsal medial.

M: Monochromatic pattern; D: Dichromatic pattern.

**Figure 1:**
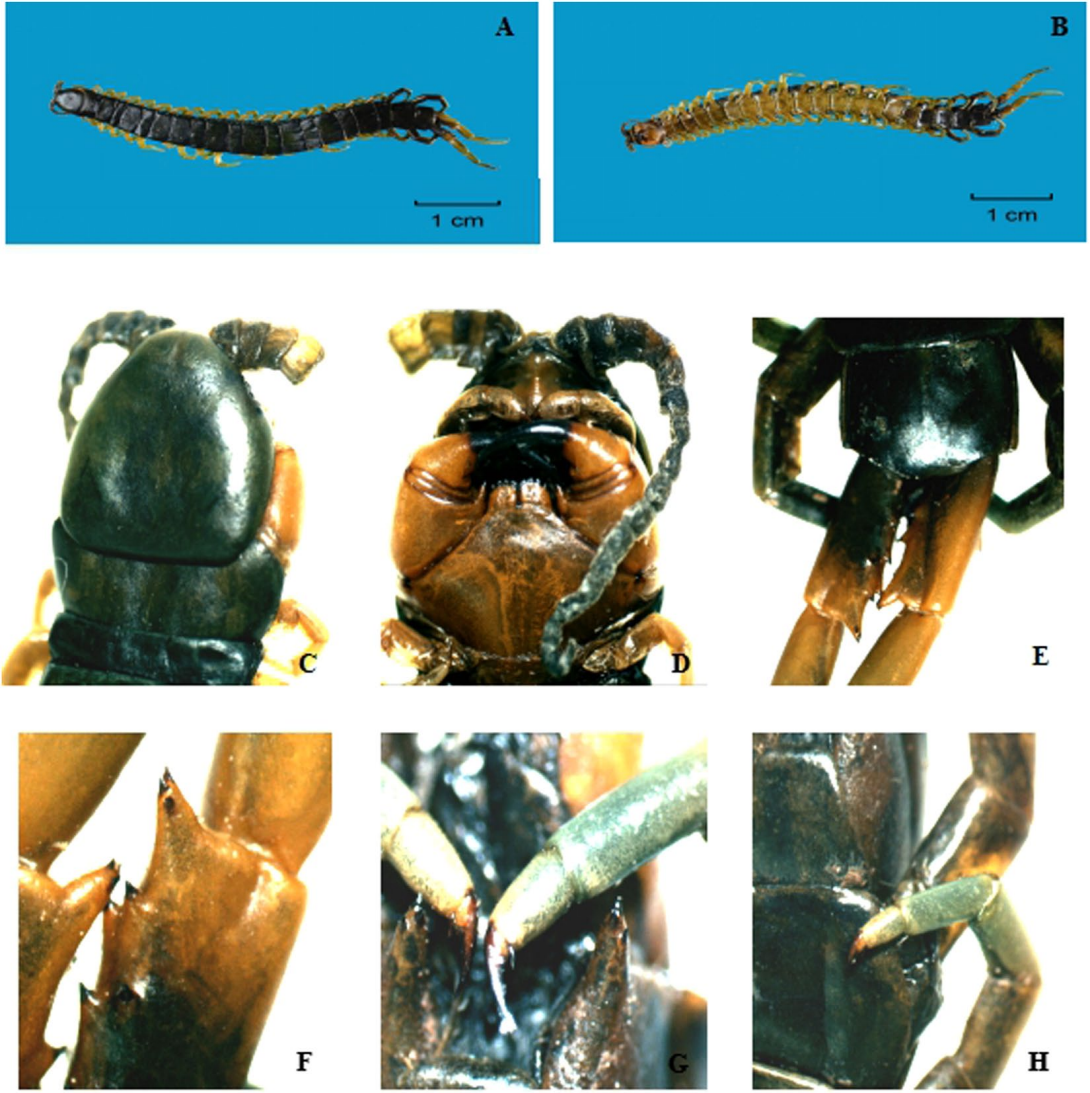
*Scolopendra lufengia*. (**A**) dorsal view of the body; (**B**) ventral view of the body; (**C**) dorsal view of the head; (**D**) ventral view of the head; (**E**) the tergite 21 and the prefemur of the ultimate legs; (**F**) spurs on the prefemur of the terminal legs and three-tipped corner spurs; (**G**) 21th leg with two accessory claws and without tarsal spur, coxopleural process with 3 corner spines; (**H**) 20th leg with tarsal spur.

Dark green-colored cephalic plate and tergites; legs are yellow at the base and gradually become green at the end; body length is less than 50 mm. There are many small pits on the head, and four ocelli on each side of the head’s front. The antenna have 18 articles, 6 of which are glabrous. The tooth plate is clearly separated into two parts, each part with 4 teeth. The paramedian sutures start on tergite 4, the complete and visible margination start at tergite 8. Tergite 21 has a complete margination without sutures or depression. Sternites 2 to 19 show nearly complete paramedian sutures. The coxopleural process is conically shaped and usually 3-tipped. A dense, small stripe-like pore area leads to the tip of the coxopleural process. All locomotory legs have 2 accessory claws, and legs 1–19 show 1 tarsal spine. The ultimate legs are strong and short; the prefemur shows 1 VL, 2 VM, 2 DM and 3 corner spines (see Fig. 1). These characteristics have never been reported in China and do not match the records of Lewis^7^. It was accordingly identified as a new species and named under the standard binomial nomenclature as *S. lufengia* sp. nov., which was registrated in Zoobank, and the registration number is urn:lsid:zoobank.org:act:AEAB0CFC-3AB6-48BB-86CB-C6A61A74D27B. The voucher specimens are preserved in the Chinese Medicine Resource Centre of Hubei University of Chinese Medicine. The holotype is registered as WGLF11-2-20151023.

Previously, researchers had mainly focused on the medicinal use of the centipedes, and some incomplete keys for these species in China were used for identification^8,32^. However, there were still many species which were not included in the keys. In this study, the morphological characteristics of 8 collected species were examined. In combination with records of 6 other species in China, i.e., *S. japonica*, *S. morsitans*, *S. amazonica*, *S. mazbii*, *S. calcarata* and *S. diaoluoensis* ^33^, we were able to systematically revise the identification key for the *Scolopendra* species in China. The key is given in Table 2.

**Table 2:** The morphyology identification Key of genus *Scolopendra* in China.

1	Spines arranged in 1-2 rows on the ventral of ultimate leg prefemur	2
—	Spines arranged in 3 rows on the ventral of ultimate leg prefemur	10
2	The color of the cephalic plate and the first tergite is nearly reddish, which is different from other tergites	3
—	The color of the cephalic plate and the first tergite is similar with other tergites	5
3	The color of the cephalic plate and the first tergite is orange-reddish, other tergites dark green. 20th leg with tarsal spur, 2 spines on the ventral lateral of the ultimate leg prefemur	***S. mutilans*** **L. Koch, 1878**
—	The color of the cephalic plate and the first tergite is reddish brown, other tergites brown. 20th leg without tarsal spur, 2-3 spines on the ventral lateral of ultimate leg prefemur	4
4	The color of the tergites is brown with red, the ultimate leg strong, male gonopods absent	***S. multidens*** **Newport, 1844**
—	The color of the tergites is brown with dirty green, the ultimate leg slender and long, male with gonopods.	***S. japonica*** **Koch, 1878**
5	The size of individual is larger, the length of the biggest is over 200 mm	6
—	The size of individual is smaller, the length is usually less than 100 mm	8
6	The ventral lateral of the ultimate leg prefemur without spines	***S. dehaani*** **Brandt, 1840**
—	The ventral lateral of the ultimate leg prefemur with 1–3 spines	7
7	Legs with orange-brown stripes, The ventral lateral of the ultimate leg prefemur with 1 spine, 20th leg without tarsal spur, male gonopods absent	***S. hainanum*** **Kronmüller, 2012**
—	The color of legs is yellow, red-brown or brown, The ventral lateral of the ultimate leg prefemur with 1–3 spines, mostly 2 spines, mostly 20th leg with tarsal spur, male with gonopods	***S. subspinipes*** **Leach, 1814**
8	The color of basal legs is yellow, gradually it become green at the end, the ventral lateral of the ultimate leg prefemur with 1 spines	***S. lufengia*** **sp.nov**.
—	The color of legs is yellow to reddish brown, the ventral lateral of the ultimate leg prefemur with 2–3 spines	9
9	The color of cephalic plate and tergites is dark brown, the basal antennal articles are yellow brown, the ventral lateral of the ultimate leg prefemur with 2 spines, and 3 corner spines on prefemur	***S. mojiangica*** **Zhang et Chi, 1989**
—	The color of cephalic plate, tergites and the basal antennal articles are dark green, the ventral lateral of the ultimate leg prefemur with 3 spines, and 2 corner spines on prefemur	***S. negrocapitis*** **Zhang et Wang, 1999**
10	The number of spines on the ventral of the ultimate leg prefemur is over 10	11
—	The number of spines on the ventral of the ultimate leg prefemur is less than 10	12
11	All legs with small setae, the ultimate leg prefemur with 9 ~ 12 VL, 11 ~ 12M, 2-3 VM spines, 1–21 legs with tarsal spur	***S. calcarata*** **Porat, 1876**
—	The leg without hair, the ultimate leg prefemur with 3 VL, 4 M, 9 VM spines, 21th leg without tarsal spur	***S. diaoluoensis*** **Z.S.Song, 2004**
12	The ultimate leg prefemur with 2 VL, 2 M, 2 VM spines, the central of cephalic plate with longitudinal suture, the end edge of tergites with dark green stripes, antennal with 17 articles	***S. mazbii*** **Gravely, 1912**
—	The sections of the ultimate leg with ridgy edge, the ultimate leg prefemur with 3 VL, 3 M, 3 VM spines, the central of cephalic plate without suture, the end edge of tergites with dark green stripes, antennal with 18 articles	13
13	20th leg without tarsal spur	***S. morsitans*** **Linnaeus, 1758**
—	20th leg with tarsal spur	***S. amazonica*** **Buecherl, 1946**

### Morphometric analysis

**Figure 2:**
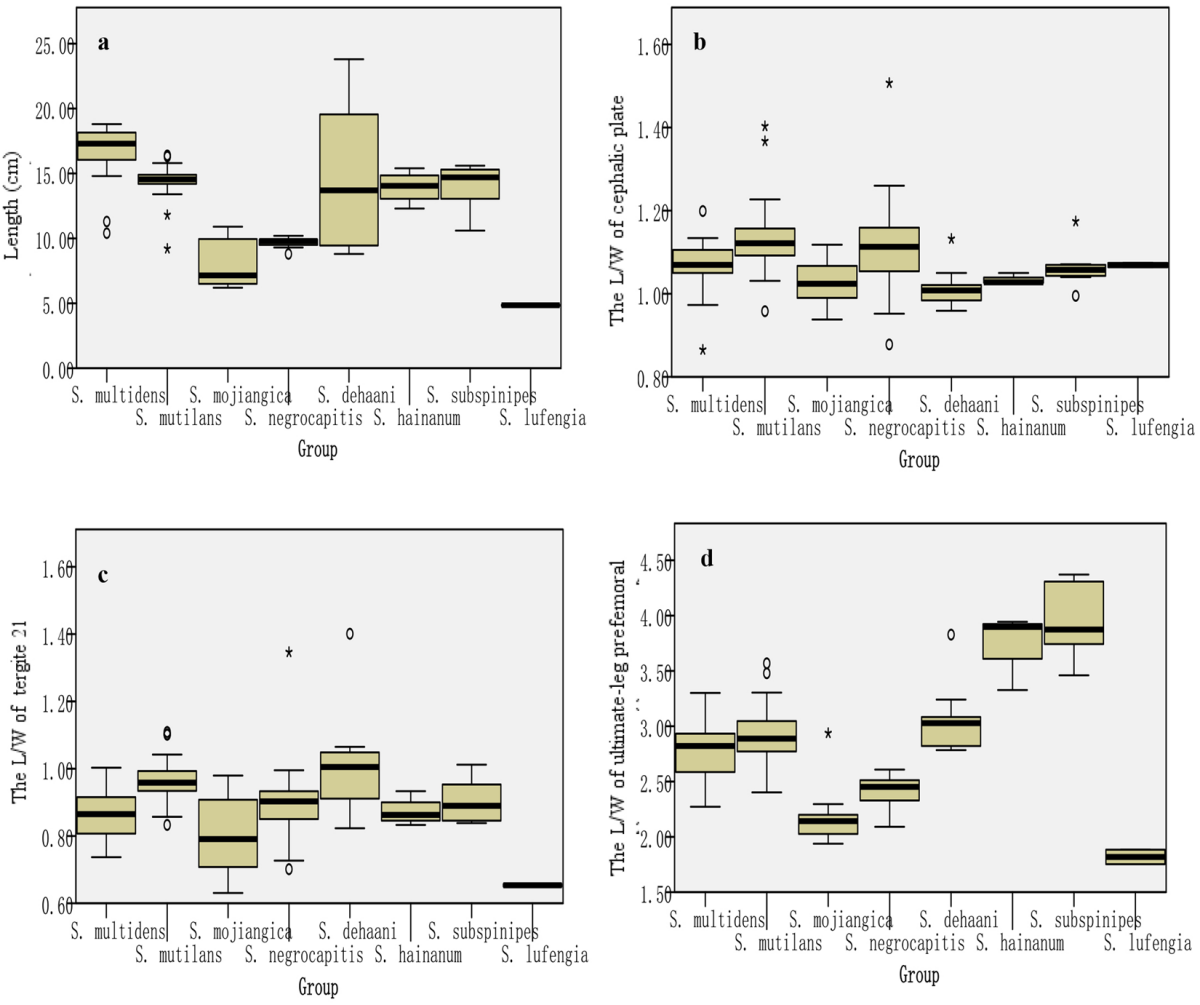
Shape variation in four characteristics of 8 *Scolopendra* species in China. (**a**) The variation ranges of body length among species. (**b**) The cephalic plate L/W ratio of species. (**c**) The tergite 21 L/W ratio of species. (**d**) The ultimate leg prefemur L/W ratio of species.

Overall, 124 representative specimens from eight species were selected for analysis. The samples were as follows: 42 specimens of *S. mutilans*, 25 of *S. multidens*, 15 of *S. dehaani*, 16 of *S. mojiangica*, 13 of *S. negrocapitis*, 8 of *S. subspinipes*, 4 of *S. hainanum* and 2 of *S. lufengia* sp.nov. The length and width of four parts that were measured in previous morphometric studies of *Scolopendra* ^2,9^, including the body, cephalic plate, tergite 21 and ultimate leg prefemur, were likewise measured in our study. The body length and the length-width ratios of the cephalic plate, tergite 21, and prefemur of ultimate leg were obtained. The shape variation was analyzed using the Kruskal-Wallis test in SPSS Statistics Version 23. The results are given in Fig. 2. Based on the statistical results, there are significant differences among groups of the four characteristics for *P* < 0.05 on the whole.

### Body length

The length ranges from 4.80 cm to 23.80 cm, the total mean is 13.36 cm, and the mean for each species is 16.64, 14.50, 7.96, 9.66, 14.52, 13.95, 14.04 and 4.85 cm respectively (see Fig. 2a). There are significant differences between *S. mojiangica* and the other four species of *S. multidens*, *S. mutilans*, *S. dehaani* and *S. subspinipes* (*P* = 0.000 to 0.025, *P* < 0.05); *S. mutilans* has significant difference from *S. multidens* and *S. negrocapitis* (*P* = 0.003 to 0.035, *P* < 0.05); and *S. multidens* has significant difference from *S. negrocapitis and S. lufengia* (*P* = 0.000 to 0.005, *P* < 0.05). Other species have no significant difference (*P* > 0.05). According to the means of the measured data, the species can be divided into two groups, namely, large- or small-size species. The large-size group mainly contains *S. multidens*, *S. dehaani*, *S. hainanum* and *S. subspinipes*. Their mean lengths are more than the total mean of 13.36 cm. The small-size group includes *S. mojiangica*, *S. negrocapitis* and *S. lufengi*a, and their mean lengths are less than 13.36 cm. Although the measured mean length of *S. mutilans* is more than 13.36 cm, its length is usually between 10 and 13 cm, for few individuals are longer than 13 cm, thus *S. mutilans* is treated as a small-size species.

### L/W ratio of cephalic plate

The length-width ratio of the cephalic plate ranges from 0.87 to 1.51. There is significant difference between *S. dehaani* and *S. mutilans* or *S. negrocapitis* (*P* = 0.000 to 0.006, *P* < 0.05), and between *S. mutilans* and *S. mojiangica* (*P* = 0.000, *P* < 0.05). However, there is no significant difference among the others (*P* > 0.05) (see Fig. 2b).

### L/W ratio of tergite 21

The tergite 21 length-width ratios range from 0.63 to 1.40. *S. mutilans* is significantly different from *S. mojiangica*, *S. multidens and S. lufengia* (*P* = 0.000 to 0.050, *P* < 0.05); *S. dehaani* is significantly different from *S. multidens, S. mojiangica and S. lufengia* (*P* = 0.001 to 0.047, *P* < 0.05). Other species do not show significant difference (see Fig. 2c).

### L/W ratio of prefemur of ultimate leg

The prefemur of ultimate leg length-width ratios of these species range from 1.75 to 4.37. There is significant difference between *S. mojiangica* and other five species of *S. multidens, S. dehaani, S. mutilans*, *S. subspinipes* and *S. hainanum* (*P* = 0.000 to 0.019, *P* < 0.05); *S. negrocapitis* has significant difference from four species of *S. dehaani, S. mutilans*, *S. subspinipes* and *S. hainanum* (*P* = 0.000 to 0.044, *P* < 0.05); *S. lufengia* aslo shows significant difference from *S. subspinipes* and *S. hainanum* (*P* = 0.001 to 0.008, *P* < 0.05)*. S. mutilans* has significant difference with *S. subspinipes* (*P* = 0.006, *P* < 0.05). There is no significant difference among the others (*P* > 0.05). As the L/W ratios are showed in Fig. 2d, *S. lufengia* and *S. mojiangica* have a stubby ultimate leg prefemur, their L/W ratios are about two, and those of *S. multidens*, *S. mutilans* and *S. dehaani* are three, whereas *S. subspinipes* and *S. hainanum* have slender ultimate legs, for their L/W ratios are approximate four.

### Molecular analysis

#### Sequence annotation

A total of 59 raw nucleotide sequences from partial gene targets for COI, representing 8 nominal species, were successfully amplified and sequenced. All raw sequences were verified with other available Scolopendromorph sequences in GenBank using the BLASTn algorithm. The results showed that all the sequences belonged to the homologous sequences of the genus *Scolopendra*, and the outgroup contamination did not affect the genomic DNA. The final aligned sequences obtained by sequence editing and the alignment program consisted of 647 bp. The G+C content is 29.9% ~ 38.0%. The contents of A+T are obviously higher than those of G+C. The average percentages of the G+C content of species are given in Table 3. The sequences of COI gene fragments consists of 327 variable sites and 320 conservative sites. Corrected genetic distances were calculated by the Kimura-2-Parameter (K2P) model for DNA sequence alignment. The intraspecific maximum K2P distance is 0.000 to 0.129, and the interspecific minimum K2P distance is 0.000 to 0.187. The intraspecific maximum distance of each species is less than the interspecific minimum distance. The interspecific and intraspecific genetic distances are summarized in Table 4.

**Table 3:** The average percentage of G + C in 12 *Scolopendra* species.

taxon	percentage	taxon	percentage
*S. mutilans*	29.9	*S. hainanum*	33.3
*S. multidens*	36.7	*S. morsitans*	34.9
*S. dehaani*	33.3	*S. japonica*	38.0
*S. subspinipes*	33.3	*S. amazonica*	33.9
*S. mojiangica*	37.1	*S. calcarata*	37.8
*S. negrocapitis*	37.0	*S. lufengia*	36.1

**Table 4:** The genetic distance calculated by K2P model for 12 *Scolopendra* species.

group	taxon	1	2	3	4	5	6	7	8	9	10	11	12	intraspecific max distance
1	*S.mutilans*													0.090
2	*S.multidens*	0.195												0.049
3	*S.dehaani*	0.129	0.152											0.079
4	*S. subspinipes*	0.211	0.219	0.192										0.000
5	*S. hainanum*	0.211	0.219	0.192	0.000									0.000
6	*S.mojiangica*	0.183	0.145	0.163	0.241	0.241								0.002
7	*S.negrocapitis*	0.197	0.139	0.156	0.222	0.222	0.121							0.047
8	*S. japonica*	0.202	0.161	0.195	0.224	0.224	0.129	0.085						0.004
9	*S. morsitans*	0.188	0.197	0.176	0.262	0.262	0.195	0.202	0.231					0.129
10	*S. amazonica*	0.178	0.190	0.195	0.251	0.251	0.223	0.224	0.224	0.144				0.106
11	*S.calcarata*	0.230	0.211	0.197	0.251	0.251	0.231	0.187	0.208	0.261	0.221			0.085
12	*S. lufengia*	0.195	0.152	0.154	0.258	0.258	0.165	0.163	0.170	0.226	0.216	0.231	—	—

#### Phylogenetic analysis

**Figure 3:**
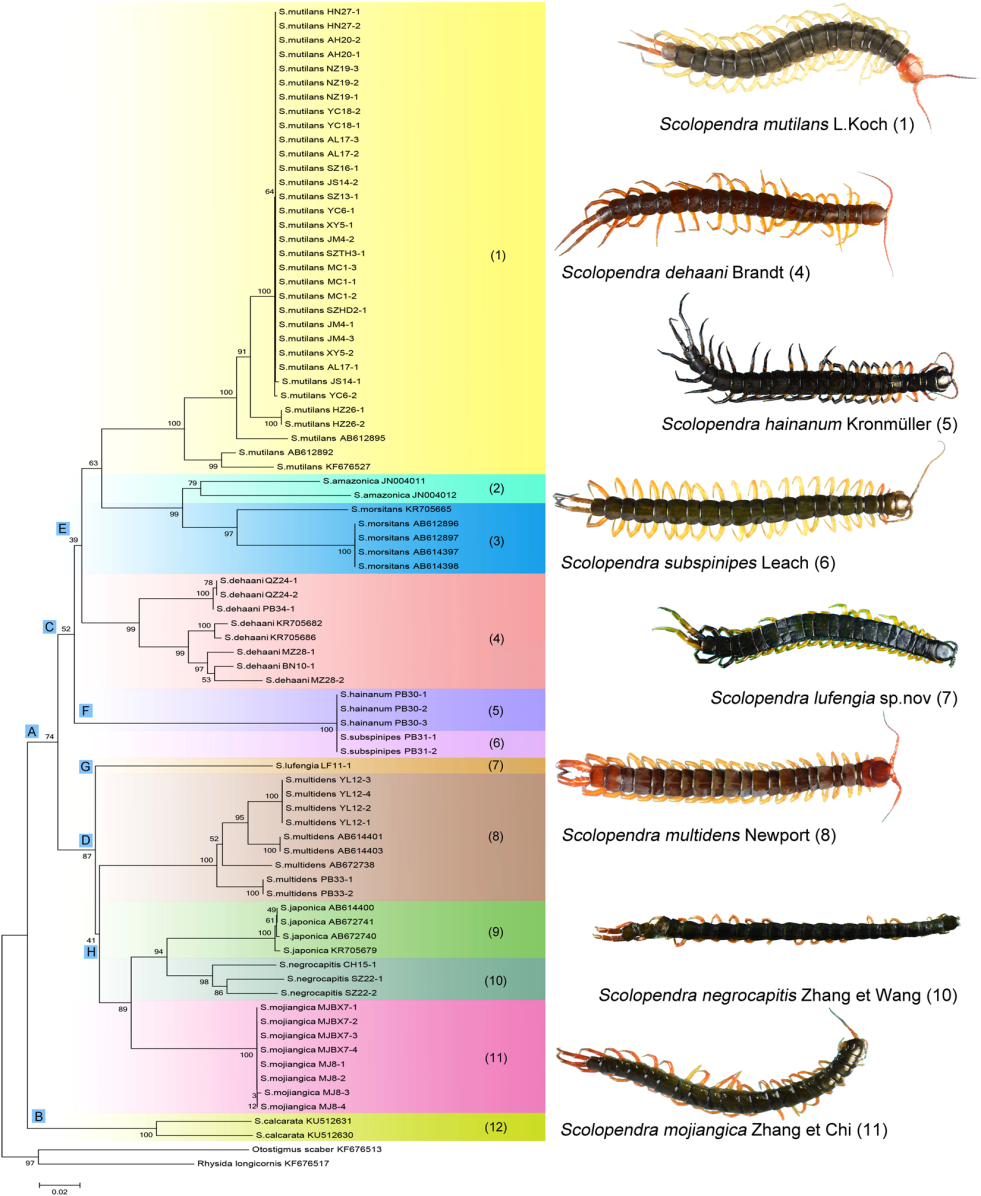
Neighbor-joining analysis of the genus *Scolopendra* in China. Relationships among *Scolopendra* and the outgroup were indicated in neighbor-joining (NJ) tree for the COI partial gene analyses. The coloration bars on the tree represent the genetic affinities relative to morphological identification in species.

**Figure 4:**
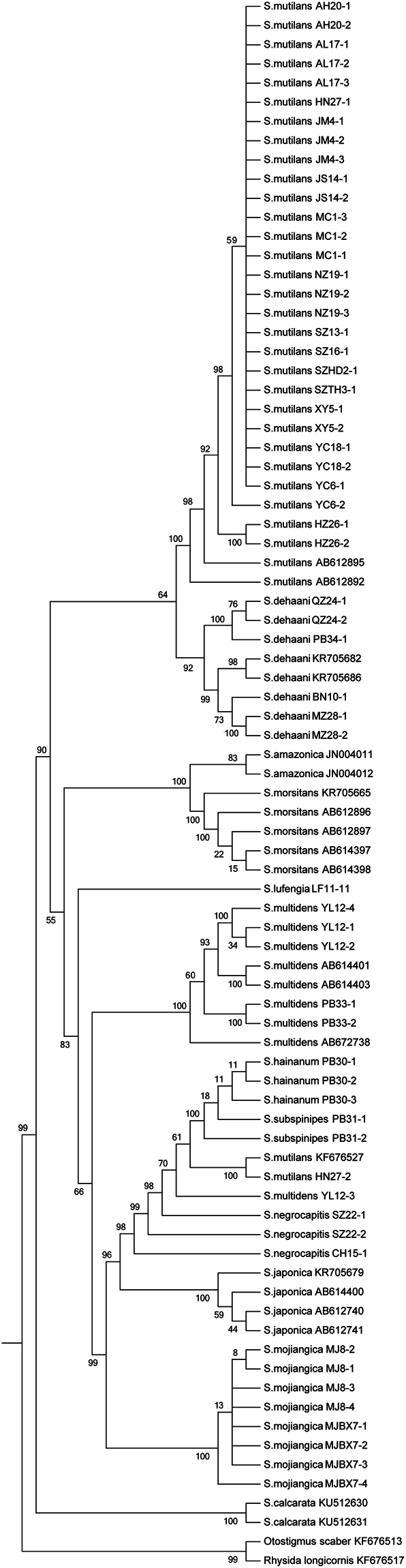
Maximum likelihood analysis of the genus *Scolopendra* in China. Relationships among *Scolopendra* and the outgroup were indicated in Maximum likelihood (ML) tree for the COI partial gene analysis. The likelihood-based analyses were performed with 1,000 bootstrap replicates, and the node values were shown as percentages.

Eighty sequences of 12 *Scolopendra* species (including 59 extracted sequences and 21 downloaded sequences) and two downloaded sequences of the scolopendrid subfamily Otostigminae, *Otostigmus scaber* Porat, 1876 and *Rhysida longicornis* Pocock, 1891 were used to construct the phylogenetic tree. Two optimality criteria of neighbor-joining (NJ) and maximum likelihood (ML) were applied for analysis. In the NJ tree, the members of *Scolopendra* are divided into two groups (see Fig. 3: clade A and clade B). The first group (clade A) is subdivided into two clades (clade C and clade D). Clade C is further divided into clade E and clade F. Clade E is mainly composed of *S. mutilans, S. amazonica*, *S. morsitans* and *S. dehaani*, whereas clade F includes *S. subspinipes* and *S. hainanum*. Clade D is further divided into clade G and clade H. Clade G is a single branch of *S. lufengia* sp. nov., whereas clade H consists of *S. multidens*, *S. japonica*, *S. negrocapitis* and *S. mojinagica*. The other group, clade B, is the species *S. calcarata*. In the topological structure, 10 morphological species of the genus *Scolopendra* are clustered into separate branches, and the taxonomy is supported by molecular data. The other two species, *S. subspinipes* and *S. hainanum*, are clustered into one branch together. They could not be differentiated from one another, and these were inconsistent with the morphological taxonomy^9^. In the ML tree, the structure shows separated groups (see Fig. 4), except *S. subspinipes* and *S. hainanum* overlaps with two sequences of *S. mutilans* and one sequence of *S. multidens* which may be suspicious sequences. The groups of *S. amazonica*, *S. morsitans*, *S. subspinipes* and *S. hainanum* are divided into different clades compared to the NJ tree. The putative species of *S. lufengia* sp. nov. is still a separate branch.

## Discussion

Scolopendromorpha are widely distributed in the world, especially in tropical and subtropical territories. As the southern China is one of the world’s biodiversity hotspots, there is an abundance of biological species, including centipedes^8^. However, comprehensive investigations of the genus *Scolopendra* in China have seldom been carried out, and the exact species and distribution range are still unknown. In our survey of many regions of China, we found that the animals of Scolopendromorpha are widely distributed across the country, but the species of the genus *Scolopendra* are mainly confined to the southern region of the Qinling-Huaihe isotherm (which is also the boundary between north and south China). Each species lives in relatively fixed distribution areas and under suitable geographical climate conditions^8^.

In recent years, many new species had been found in China, such as *S. negrocapitis* and *S. mojiangica* ^34,35^. Many former subspecies, i.e, *S. multidens*, *S. dehaani* and *S. japonica*, were also elevated as valid species^8,9,32^. Although there are obviously differences in morphological characteristics between *S. mutilans* and *S. subspinipes*, Siriwut^1^ failed to distinguish these two species in the phylogenetic tree. However, in our study, they clustered into two different branches in the tree, and the result is consistent with report of Vahtera^36^, which supports the status of separate species. Previously*, S. subspinipes* was considered to be a variable species widely distributed in the southern area, Kronmüller^9^ explored the differences among individuals in morphology, and then *S. hainanum* was separated from *S. subspinipes* as a new species. Nevertheless, these two morph-species are not supported by our molecular information and morphometric data. The samples collected from Lufeng, Yunnan, have specific morphological characteristics as described above, which are obviously different from those of other species in China. The morphometric data show that this species has a small body length and strong ultimate leg prefemur with a ratio of approximately twice the length to width. The molecular analysis also indicates its independent taxa status, which is resolved as a separate branch in the phylogenetic tree. Its geographic distribution is near to that of *S. mojiangica*, but it is obviously different from *S. mojiangica* in both morphology and molecular features. As a result, we assumed it to be a new species and named it *S. lufengia* sp. nov. According to our description and sporadic reports^8,32–34^, to date, there are 14 species of *Scolopendra* distributed in China.

**Figure 5:**
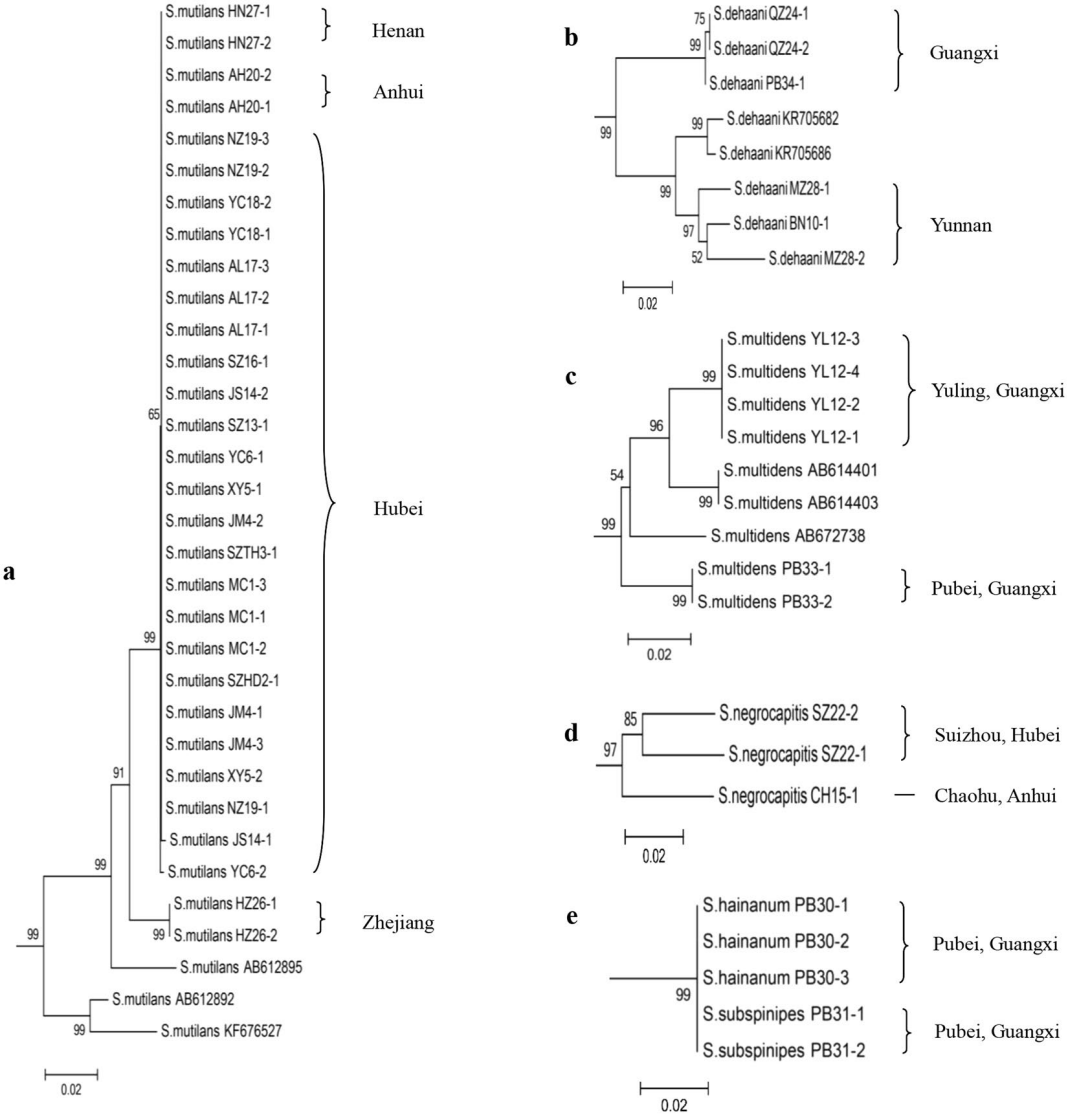
Phylogenetic relationship of the genus *Scolopendra* based on genetic structure among its populations relative to regional distribution in China. (**a**) The population of *S. mutilans* from Hubei and adjacent areas gathered into a branch while samples from Zhejiang clustered into another branch in the structure. (**b**) The genetic structure of *S. dehaani* indicates that its populations sampled from Guangxi and Yunnan gathered into a different branch. (**c**) The population of *S. multidens* sampled from Yulin and Pubei of Guangxi gathered into a different branch. (**d**) Significant difference of *S. negrocapitis* in the Phylogenetic tree caused by distribution. (**e**) The two species of *S. subspinipes* and *S. hainanum* cluster into a single branch which shows no genetic difference.

Previously, morphological examination was the main approach applied for taxonomy of the genus *Scolopendra*. However, there is much debate around the taxonomic status of some species, especially in terms of the taxonomy of the former *S. subspinipes* complex and *S. morsitans* complex. In our study, an integrated method of morphology-molecular analysis has been used to validate the taxonomy. Based on the morphological identification, the characteristics of the collected samples were redescribed. Combined with previous reports, the identification key was revised to show the main differences among *Scolopendra* species in China. The morphometric analysis made the subjective characteristics digitized and more intuitive, which further highlighted the morphological variations among species, especially in the body length and the prefemur of ultimate leg. The COI barcoding both in NJ and ML analyses further confirmed the determination, and it provided a new approach to solve these difficulties in identification by using the genetic distance and phylogenetic trees, from which 10 of the 12 putative species were successfully classified. There are deficiencies in identifying damaged or powder samples by morphological methods, difficulties also existed in classifying species that are closely related using molecular methods, but the integrated approach can make these two methods complement each other, which showed good results in our identification of the *Scolopendra* species. In this way, we believe that the integrated approach gives evidence for the validation of the *Scolopendra* species in China to be separate species; thus, the former subspecies of *S. subspinipes* and *S. morsitans* in China are reasonably valid species. The morphological characteristics of *S. lufengia* do not match those of other *Scolopendra* species, and it can also be confirmed by molecular data to be separate taxon. In contrast, the characteristics of *S. hainanum* match the description given by Kronmüller^9^ (see Table 1). However, the molecular data suggests that there is no obvious difference between *S. hainanum* and *S. subspinipes* in either the genetic distance or the phylogenetic tree, so these two morph-species would be considered one molecular-species for clustering into a single branch on the tree. We speculate that there would be a close relationship between these species, and that the unapparent differences of genetic distance in COI gene fragments from our limited samples and data from the same collected area of Pubei, Guangxi (Fig. 5e) would not be sufficient to be reflected on the tree. Thus, we consider the taxonomic status of *S. hainanum* to be still undetermined, and taxonomy for these two morph-species still warrants further study. In our study, the genetic relationships among most species are consistant in the NJ and ML trees, but the species of *S. amazonica*, *S. morsitans*, *S. subspinipes* and *S. hainanum* are clustered into different clades.

Meanwhile, although the medical value of *Scolopendra* has gradually been recognized, the high risk of toxicity to humans is an inevitable topic^17,37^. Because there is difference in the toxicity and pharmacological effect between species^18^, accurately identifying *Scolopendra* species will help to reduce the risk, and their medicinal properties would be fully utilized. In this study, the authenticity of the *Scolopendra* species in China can be verified using the integrated method of morphology identification and COI barcoding, and *S. mutilans* can be distinguished accurately from the local medicinal species or other commonly confused species in markets.

In morphological examination, characteristics are usually used to evaluate identification and taxonomy. However, in fact, the importance of the characteristics in evaluation are not always equivalent, because there are flexible features, such as coloration which is one of the most variable characteristics. Usually the species are divided into monochromatic or dichromatic pattern according to the color of cephalic plate and tergites. Most individuals of *S. dehaani* have a monochromatic pattern for the brown body, but some of them also have reddish cephalic plates and brown or entirely black tergites, comprising a dichromatic pattern. Even the same color pattern maybe displays difference. *S. subspinipes* has a monochromatic pattern, and the coloration has a large range, which varies from reddish brown to blackish. The yellow legs of *S. mutilans* from Hubei province and the red legs from Zhejiang province are another obvious example in coloration. In immature stages or at ecdysis, the color is also relatively duller than in adults. The margination or paramedian suture on tergites and sternites is another unstable characteristic.The first tergite with suture or margination is occasionally inconspicuous in individuals, and can overlap with other related species. Abnormal structures will also cause errors in identification and taxonomy. Sometimes smaller or special regenerations are found in a damaged leg or antenna, which are obviously different from the protogenetic legs or antennae. Thus, it is not sufficient to differentiate species just based on these variable characteristics. However, some stable and specific features should be chosed as diagnostic characteristics. For example, *S. mutilans* has tarsal spurs on 20 legs, whereas the 20 legs of *S. multidens* lack tarsal spurs; the gonopods of *S. multidens* and *S. hainanun* are not visible; and ventral lateral spines are lacking on the ultimate leg prefemur of *S. dehaani*. In our morphometric study, four features are used to show the shape variation. In light of the body length and the length-width ratio of the ultimate leg prefemur, the samples can be easily divided to different types. Hence, we supposed that these critical and stable characteristics, including antennae, gonopods, tarsal spurs on 20th leg and spines on the ultimate leg prefemur, as well as the length and length-width ratio of the ultimate leg prefemur (see Table 1 and Fig. 2), should be regarded as important taxonomical characteristics, which would play a very important role in identification and taxonomy.

Reports have shown that some *Scolopendra* species have different patterns, in either external morphology or genetic materials, that are caused by their geographic environments. *S. dehaani* in Southeast Asia has five color patterns, and they are clustered into five small branches in the phylogenetic tree^2^. In our study, we found that the color of the legs in most individuals of *S. mutilans* was yellow in Hubei province, but red in Zhejiang province. In the phylogenetic tree, samples of *S. mutilans* from Hubei and the adjacent areas of Henan and Anhui are all clustered into one branch, whereas samples from Zhejiang are united into another branch (Fig. 5a). These conditions caused by distribution also exist in *S. multidens* and *S. negrocapitis*. The samples of *S. multidens* collected from Yulin and Pubei are clustered into separate branches (Fig. 5c), and samples of *S. negrocapitis* collected from Suizhou of Hubei and Chaohu of Anhui are also clustered into two separate branches (Fig. 5d). *S. dehaani* collected from Yunnan and Guangxi further confirmed these patterns (Fig. 5b). We consider that the geographical and ecological environments, such as mountains or plains, temperature and humidity, etc, might play an important role in difference of morphology or genetic material.

In our study, although as many as 39 batches of samples were collected, there are still limitations. First, it is difficult to obtain enough samples from all regions, some species can not be collected successfully, or the sample size of some species is small (e.g., only two specimen of *S. lufengia* were collected). In addition, certain samples were of poor quality after being stored for a long time, so their sequences could not be extracted during our molecular analysis. These factors may lead to uncertainty in the results. In future, further investigations and more representative samples would be needed to perfect the result and bring more accuracy to the identification and taxonomy of *Scolopendra* in China, especially in the putative species of *S. lufengia*. But this integrated method of morphology-molecular analysis would also provide a reference for the study of other species and medicinal materials.

## Materials and Methods

### Sample materials

A total of 39 batch samples were collected from the natural field during the course of surveys in southern China, commodity markets or companies since 2015. The sample information is given in Table 5. The living specimens were relaxed with 50% ethanol for 10~20 min and then transferred into 70% ethanol to settle their posture for photography. The samples for molecular analysis were kept in absolute ethanol at −40 °C. The others were kept in conditions below −20 °C. All samples are housed in Hubei University of Chinese Medicine, Wuhan, China.

**Table 5:** List of voucher specimens of *Scolopendra* species and sequences for DNA analysis.

Species	Locality & company	Batch number	Collection time	Specimens for DNA	GenBank number
*S. mutilans* L.Koch	Macheng, Hubei	MC1	2015.4.18	MC1-1∼3	KX525589, KX525590, KX525591
	Suizhou Hedian, Hubei	SZHD2	2015.4.21	SZHD2-1	KX525592
	Suizhou Huantan, Hubei	SZHT3	2015.4.21	SZHT3-1	KX525593
	Jinmen, Hubei	JM4	2015.5.15	JM4-1∼3	KX525594, KX525595, KX525596
	Xiangyang, Hubei	XY5	2015.6.2	XY5-1∼2	KX525597, KX525598
	Yichang, Hubei	YC6	2015.6.3	YC6-1∼2	KX525599, KX525600
	Suizhou, Hubei	SZ13	2015.6.24	SZ13-1	KX525601
	Jinshan, Hubei	JS14	2015.7.22	JS14-1∼2	KX525602, KX525603
	Suizhou, Hubei	SZ16	2015.8.29	SZ16-1	KX525604
	Anlu, Hubei	AL17	2015.9.7	AL17-1∼3	KX525605, KX525606, KX525607
	Yichang, Hubei	YC18	2015.9.14	YC18-1∼2	KX525608, KX525609
	Nanzhang, Hubei	NZ19	2015.9.18	NZ19-1∼3	KX525610, KX525611, KX525612
	Anhui	AH20	2015.6.15	AH20-1∼2	KX525613, KX525614
	Huqingyu Tang Pharmacy Co., Ltd. (Hangzhou, Zhejiang)	HZ26	2015.11.23	HZ26-1∼2	KX525615, KX525616
	Hangzhou Fang Huichun Tang Co., Ltd. (From Henan)	HN27	2015.11.28	HN27-1∼2	KX525617, KX525618
	Macheng, Hubei	MC32	2016.5.1	/	/
					AB612898, AB612892, KF676527
*S. multidens* Newport	Yulin, Guangxi	YL12	2015.6.24	YL12-1∼4	KX525619, KX525620, KX525621, KX525622
	Kunming Chrysanthemum Garden Chinese medicine market (From Guangxi)	GX21	2015.6.15	/	/
	Guangxi	GX29	2015.12.1	/	/
	Pubei, Guangxi	PB33	2016.5.10	PB33-1∼2	KX525623, KX525624
	Guangxi	GX39	2016.8.8	/	/
					AB614401, AB614403, AB672738
*S. mojiangica* Zhang et Chi	Mojiang Bixi, Yunnan	MJBX7	2015.6.13	MJBX7-1∼4	KX525636, KX525637, KX525638, KX525639
	Mojiang,Yunnan	MJ8	2015.6.12	MJ8-1∼4	KX525640, KX525641, KX525642, KX525643
*S. negrocapitis* Zhang et Wang	Chaohu, Anhui	CH15	2015.8.8	CH15-1	KX525644
	Suizhou, Hubei	SZ22	2015.11.14	SZ22-1∼2	KX525645, KX525646
*S. dehaani* Brandt	Xishuangbanna, Yunnan	BN9	2015.6.13	/	/
	Xishuangbanna, Yunnan	BN10	2015.6.14	BN10-1	KX525625
	Qinzhou, Guangxi	QZ24	2015.11.23	QZ24-1∼2	KX525626, KX525627
	Qingdao Hongde Sheng Prepration Co. Ltd. (From Mengzi, Yunnan)	MZ28	2015.12.4	MZ28-1∼2	KX525628, KX525629
	Pubei, Guangxi	PB34	2016.5.10	PB34-1	KX525630
	Guangxi	GX38	2016.8.8	/	/
					KR705682, KR705686
*S. subspinipes* Leach	Qinzhou, Guangxi	QZ25	2015.11.23	/	/
	Pubei, Guangxi	PB31	2016.4.26	PB31-1∼2	KX525634, KX525635
	Pubei, Guangxi	PB35	2016.5.10	/	/
	Pubei, Guangxi	PB36	2016.5.10	/	/
	Pubei, Guangxi	PB37	2016.5.10	/	/
*S.hainanum* Kronmüller	Qinzhou, Guangxi	QZ23	2015.11.23	/	/
	Pubei, Guangxi	PB30	2016.4.26	PB30-1∼3	KX525631, KX525632, KX525633
*S. lufengia* sp.nov.	Lufeng, Yunnan	LF11	2015.10.23	LF11-1	KX525647
*S. japonica* L.Koch					KR705679, AB614400, AB672740, AB672741
*S. morsitans* Linnaeus					KR705665, AB612896, AB612897, AB614397, AB614398
*S. amazonica* buecherl					JN004011, JN004012
*S. calcarata* Porat					KU512631, KU512630
*Otostigmus scaber* Porat					KF676513
*Rhysida longicornis* Pocock					KF676517

### Morphological identification

Based on classical characteristics recorded by Siriwut^2^, Kang^8^, Kronmüller^9^, Song^33^, Zhang^34^, and Chao^38^, *et al*., the identification of the samples was performed. A series of diagnostic characteristics was involved in the identification, and the details shown as follow, coloration, shape size, number of antennal articles, and those that are sparsely hirsute or glabrous, number of teeth, spines on the coxopleuron, prefemur spines arrangement on the ultimate legs, presence or absence of tarsal spurs on legs 19 and 20, and presence or absence of a gonopod on the first genital segment of the male. The photos were taken with a Nikon D7000 digital camera assembled with a Nikonlens, and the fine features were observed using an Olympus optical stereomicroscope equipped with an imaging system (Olympus, Japan). All samples were classified into nominal species, and the identification key of species in China was revised.

### Additional morphometrics

The representative specimens were chosen randomly from the samples of nominal species to avoid personal bias. In light of previous literatures, some characteristics have important value in identification and taxonomy^2,9^. The typical morphological features of four parts, including the body, cephalic plate, tergite 21 and ultimate leg prefemur, were chosed for this analysis. The length and width of these four parts were measured with a vernier caliper; and the length-width ratios of the cephalic plate, tergite 21 and ultimate leg prefemur were calculated. The data was analyzed using the SPSS Statistics Version 23 software. The Kruskal-Wallis test was used to determine significant difference. A *P* value less than 0.05 was considered to indicate statistical significance.

### DNA extraction, amplification and sequencing

40mg separated tissue from the locomotory legs of samples was disinfected with 75% ethanol and then dissected. The blood/cell/tissue TIANamp Genomic DNA Kit (Tiangen Biotech Co., China) was applied to extract total DNA according to the instructions. The PCR mixture consisted of the following: 2.0 μL of DNA template, 1.0 μL of forward and reverse primers, 12.5 μL of 2×Taq PCR Mix and 8.5 μL of ddH_2_O. COI gene fragments were chosen for detection, and the universal primer sequences used were 5′-GGTCAACAAATCATAAAGATATTGG-3′ (LCO1490, forward) and 5′-TAAACTTCAGGGTGACCAAAAAATCA-3′ (HCO2198, reverse)^39^. All PCR mixtures were activated using a PCR analyzer (Prime 5G, Techne British Co.). The COI gene amplification was performed under conditions of PCR reactions cycled at 94 °C for 1 min as an initial step, followed by 5 cycles of 94 °C for 1 min in a denaturation step, 45 °C for 1.5 min in an annealing step, and 72 °C for 1.5 min in an extension step. This process was followed by 35 cycles of 94 °C for 1 min in the denaturation step, an annealing step at 50 °C for 1.5 min, 72 °C for 1 min in an extension step, and then a final extension step at 72 °C for 5 min. The PCR cycler was programmed at a holding temperature of 4 °C as the final step. The PCR products were detected on 1% (w/v) agarose gel electrophoresis in a 0.5×TBE buffer. The fluorescence of PCR bands were enhanced with SYBR Safe illuminant and observed under UV light. The PCR products were directly cycle-sequenced using the Sanger method and original amplification primers at Sangon Biotech (Wuhan) Co., Ltd.

### Phylogenetic reconstruction

DNA sequences were assembled in CodonCode Aligner V5.1.5 (CodonCode Co., USA). Double strand sequence comparisons were made using a shadow pairwise alignment function analysis to detect missing sites and gaps in nucleotide sequences based on the chromatograms for each sequence sample. The sequences (including the sequences from 8 collected species and 4 species from GenBank; 8 sequences of *S. mutilans*, *S. dehaani* and *S. multidens* were also from GenBank) were used to construct the phylogenetic tree. Moreover, two sequences of the scolopendrid subfamily Otostigminae from GenBank, *Otostigmus scaber* Porat, 1876 and *Rhysida longicornis* Pocock, 1891 were chosen as outgroup to root the trees. All of the final sequences were blasted in GenBank with the homologous sequences as a check to evaluate the efficiency and then submitted to GenBank. A summary of the sequences information is in Table 5. The DNA sequences were aligned using MUSCLE, and the genetic distances were computed using MEGA 6.06 according to the K2P model. The NJ and ML analysis were applied to construct phylogenetic trees. The NJ analysis was constructed in MEGA 6.06, the concatenated files were analyzed with K2P model, and the likelihood-based analyses were performed with 1,000 bootstrap replicates. Codon positions were combined as 1st+2nd+3rd+Noncoding. All positions containing gaps and missing data were eliminated. The ML analysis was conducted using PhyML 3.0 (Online execution) based on the General Time Reversible (GTR) model. Fast likelihood-based analyses were performed with 1000 bootstrap replicates, SPRs was applied for the tree search, and the starting tree was selected with BIONJ.
